# Dissecting Cis Regulation of Gene Expression in Human Metabolic Tissues

**DOI:** 10.1371/journal.pone.0023480

**Published:** 2011-08-31

**Authors:** Radu Dobrin, Danielle M. Greenawalt, Guanghui Hu, Daniel M. Kemp, Lee M. Kaplan, Eric E. Schadt, Valur Emilsson

**Affiliations:** 1 Merck Research Laboratories, Rahway, New Jersey, United States of America; 2 Merck Research Laboratories, Boston, Massachusetts, United States of America; 3 Massachusetts General Hospital, Boston, Massachusetts, United States of America; 4 Pacific Biosciences, Menlo Park, California, United States of America; 5 Icelandic Heart Association, Kopavogur, Iceland; Kyushu Institute of Technology, Japan

## Abstract

Complex diseases such as obesity and type II diabetes can result from a failure in multiple organ systems including the central nervous system and tissues involved in partitioning and disposal of nutrients. Studying the genetics of gene expression in tissues that are involved in the development of these diseases can provide insights into how these tissues interact within the context of disease. Expression quantitative trait locus (eQTL) studies identify mRNA expression changes linked to proximal genetic signals (cis eQTLs) that have been shown to affect disease. Given the high impact of recent eQTL studies, it is important to understand what role sample size and environment plays in identification of cis eQTLs. Here we show in a genotyped obese human population that the number of cis eQTLs obey precise scaling laws as a function of sample size in three profiled tissues, i.e. omental adipose, subcutaneous adipose and liver. Also, we show that genes (or transcripts) with *cis* eQTL associations detected in a small population are detected at approximately 90% rate in the largest population available for our study, indicating that genes with strong cis acting regulatory elements can be identified with relatively high confidence in smaller populations. However, by increasing the sample size we allow for better detection of weaker and more distantly located *cis*-regulatory elements. Yet, we determined that the number of tissue specific cis eQTLs saturates in a modestly sized cohort while the number of cis eQTLs common to all tissues fails to reach a maximum value. Understanding the power laws that govern the number and specificity of eQTLs detected in different tissues, will allow a better utilization of genetics of gene expression to inform the molecular mechanism underlying complex disease traits.

## Introduction

The development of high throughput technologies that allow for whole-genome profiling of genotypes, gene expression traits, proteins and metabolites in multiple tissues/samples from a single individual, can provide important clues to disease mechanisms. We have recently shown the ability to associate genetic changes to variations in mRNA levels in an obese population undergoing Roux en Y Gastric bypass surgery (RYGB) [1]. Similar approaches in all realms of biology from diabetes, obesity, cardiovascular disease, to asthma and Crohn's disease have proved to be very successful in identifying new therapeutic targets [2], [3], [4]. In recent years, numerous genome-scale technologies have been employed to explore the genetics of gene expression in human cell lines and primary tissues [2], [5], [6], [7]. These studies have generated a vast amount of quantitative data, providing us with a systems view of disease mechanisms [4], [8], [9], [10], [11], [12], [13], [14], [15].

In the present study, we explored 37,585 gene expression traits in 744 omental adipose (OA), 612 subcutaneous adipose (SA) and 569 liver samples collected from self reported Caucasian subjects undergoing RYGB where each subject was genotyped for 650,000 SNPs. Only 393 subjects had genotyping and expression profiling data available for all three tissues and were used in this analysis. Given the size of the cohort and the availability of multiple tissues from the same subject we were able to analyze the genetics of gene expression in each tissue in an attempt to understand the laws governing the number and tissue specificity of cis eQTLs detected. Our main goal was to identify the power laws relating to the number and specificity of cis eQTLs detected as a function of sample size. Understanding these relationships will allow us to better utilize the genetics of gene expression to inform on the molecular mechanism underlying complex disease.

## Results

DNA was extracted from liver samples and genotyped on Illumina 650Y genotyping arrays. RNA was extracted from OA, SA and liver samples and profiled on custom Agilent expression arrays containing 37,585 probes for gene expression traits. 393 subjects, which are self-reported Caucasians, were included in our analysis for which genotyping and gene expression data were available from all three tissues [1]. Cis eQTLs were identified using the Kruskal-Wallis non-parametric test described previously [2]. In order to reduce the number of hypotheses tested, i.e. the pair SNP-mRNAs, only the pair with the smallest p value in a 2 Megabase (Mb) window was retained, similar to the method used in previously published studies [2], [5]. A cis eQTL is thus the association between a SNP and the expression levels of a transcript, where the SNP is located within the genomic region that spans 1 Mb upstream and downstream of the transcript's start site, and with a p value below the p value threshold selected to maintain a 10% FDR. In order to avoid any confusion we will refer to the eQTLs as the transcript rather than the SNP-transcript pair, while an expression (e)SNP is the selected SNP associated with the mRNA profile of a transcript.

**Figure 1:**
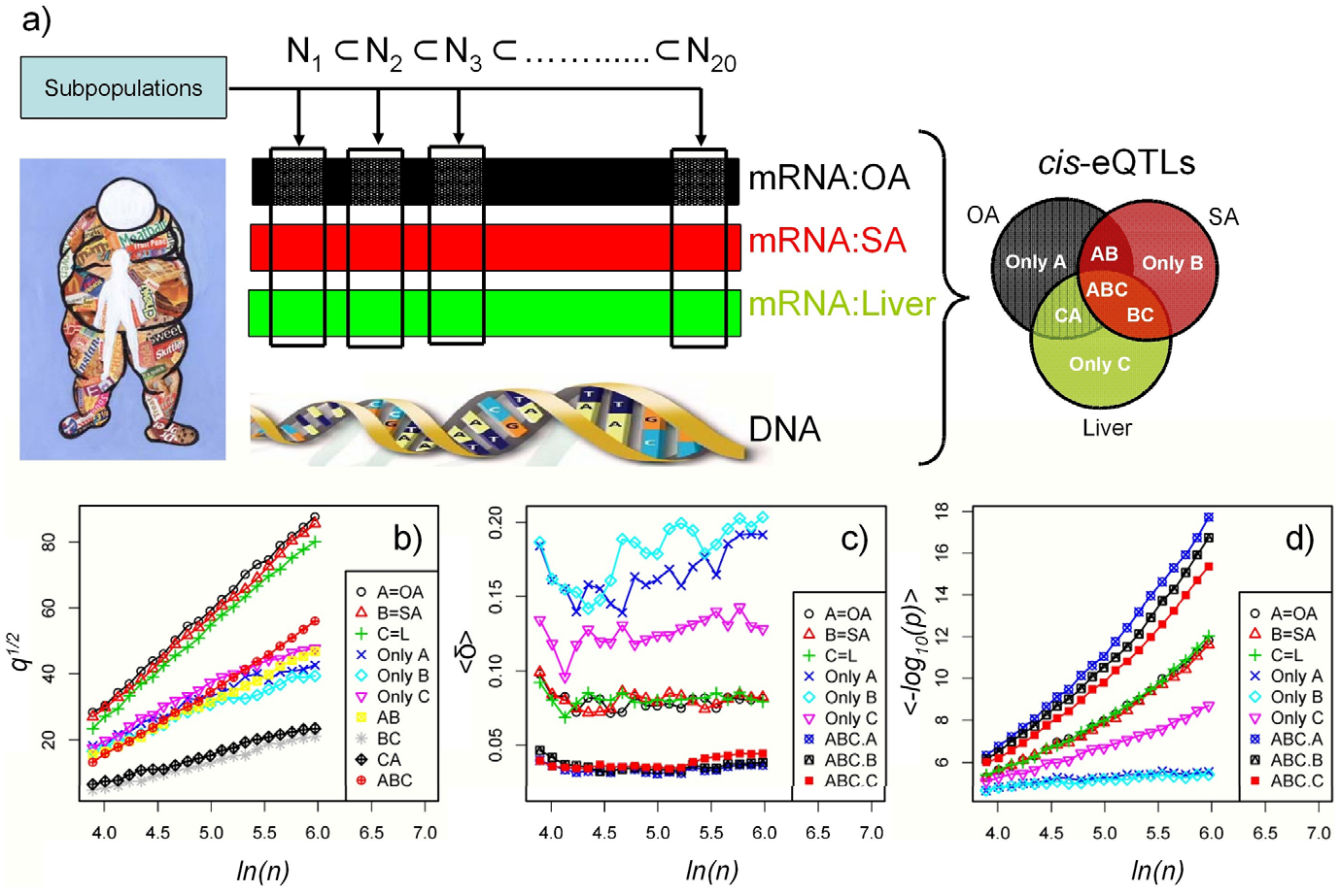
Number of cis eQTLs and their link to sub sample size. **a**) Experimental design. Starting with 393 individuals that were successfully gene expression profiled in all 3 tissues (in the figure legends A, B, C denote OA, SA and liver respectively) and genotyped, we selected 20 sub samples with sizes from 50 to 393 equally spaced on the logarithmic scale such that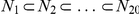. For each sub sample we identified cis eQTLs in each tissue at 10% FDR. **b**) Number of cis eQTLs *q* as function of sample size *n*. We plot 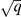 as a function of *ln(n)* and show that total number of cis eQTLs identified in each tissue is linear in this range with 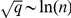
with *R^2^>0.9* (see Table 1 for linear regression coefficients). Looking at cis eQTLs across the 3 tissues we observ a tendency to plateau for cis eQTLs that are nonoverlapping (Only A, Only B and Only C) or partially overlapping in a pair of tissue (AB, BC and CA). No such behavior is observed for cis eQTLs common to all three tissues (ABC). **c**) Mean genomic distance 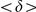 measured in Mb between the eSNP and the transcript's position. Common cis eQTLs (ABC.A, ABC.B and ABC.C) have 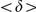 significantly smaller (Mann–Whitney *U* test p values ∼0) than any 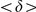 computed for the nonoverlapping cis eQTLs (Only A, Only B, and Only C). **d**) Mean association strength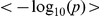. Overlapping cis eQTLs have on average a stronger association than any other sub set of cis eQTLs. Unique cis eQTLs (Only A, Only B, and Only C) are weakly associated when compared to overlapping cis eQTLs (ABC).

We generated 20 sub-samples *N_i_ i = 1*∶*20* equally spaced in the *ln*(*n*) space (where *n* = |*N*| is the size of sample *N*) by randomly removing individuals from the largest sample *N_20_* of size *n_20_* = 393 until we reached a sample of size *n_1_* = 50 (see Figure 1a for the study design and Table S1). For each sub sample we removed all SNPs that did not exceed a minor allele frequency (MAF) of 5% (the number of SNPs removed from the analysis for each sub sample is summarized in the Table S1). For each tissue and each sub-sample we then proceeded to identify the cis eQTLs and eSNPs. The sub sample generating procedure and subsequent analysis was repeated five times (see Figure S5).

Let *Q_i_* and *S_i_* be the set of cis eQTLs and eSNPs respectively identified using the population sub-sample *N_i_* with *q_i_* = |*Q_i_*| and *s_i_* = |*S_i_*| the number of unique cis eQTLs and eSNPs in each set. We found that the number of cis eQTLs precisely obeys 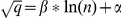 (Figure 1b) for each tissue in the analysis (see Figure S1 for a comparison of plots in normal and log-normal space). The slopes 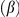 and intercepts 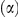 for each tissue are summarized in Table 1. Slopes for changes in total number of cis eQTLs as a function of sample size in each tissue are very close in value thus we hypothesize that the scaling laws will hold in any analysis of tissues collected from this cohort however we believe that slope could change if tissue samples were collected from a different cohort. The observed intercept change when analyzing this particular cohort, with 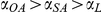
is most likely a consequence of tissue heterogeneity. We believe that the number of cis eQTLs detected in cellular homogenous tissues (such as adipose) is larger than in a more heterogeneous tissue (such as liver) explaining the differences between the intercepts in each tissue. Similar behavior was previously noted in an Icelandic population [2], [16] with 3,048 cis eQTLs detected in adipose tissue and 2,417 in whole blood from the same individuals.

**Table 1:** Extrapolated scaling laws for cis eQTLs.

Tissue	Intercept(alpha)	Slope (beta)	R^2^	Observed	Calculated
**OA**	−85.832	29.131	0.998	11,018	11,403
**SA**	−85.284	28.591	0.998	9,874	9,639
**Liver**	−83.237	27.531	0.999	8,278	8,358

We fit,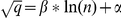 where *q* is the number of cis eQTLs and *n* sample size. The results of the linear fit for each tissue are presented below. Using this model we estimated the number of cis eQTLs detected in each tissue at maximum sample size. The calculated values approximate very well the observed numbers of cis eQTLs in each tissue.

While the number of cis eQTLs in each tissue obeys the same scaling law as described above, by looking at overlaps between cis eQTLs across the 3 tissue we identified subsets of cis eQTLs that behaved dramatically differently as a function of sample size. We classified cis eQTLs in seven groups. If A denotes OA tissue, B - SA tissue and C - liver then “Only A”, “Only B”, “Only C” represent cis eQTLs unique (i.e. nonoverlapping) in OA, SA and liver tissues, “AB”, “BC”, “CA” represent the sets of cis eQTLs overlapping in pair of tissues such as OA-SA, SA-liver and liver-OA, and “ABC” represents the set of cis eQTLs detected in all three tissues. By looking at the plots in Figure 1b we identified 2 qualitatively different behaviors for their set size as a function of sample size: set sizes for partially overlapping and nonoverlapping cis eQTLs (Only A, Only B, Only C, AB, BC and CA) show a tendency to plateau as we increase the sample size, while the number of overlapping cis eQTLs continues its steady increase at a rate that will approach 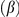 identified for single tissues as sample size increases. Using a sliding window containing consecutive data points a more qualitative picture can be obtained (see Figure S2). A decrease in slope (as we slide the window from the beginning of the plot to the end) signals a saturation tendency for the number of cis eQTLs in the set while an increase in slope signals a further acceleration. Using this approach we again see the 2 different trends observed in Figure 1b, saturation in the number cis eQTLs that are not detected (tissue specific) in all three tissues. The trend is more pronounced for cis eQTLs overlapping across less related tissues, such as OA-liver or SA-liver, while cis eQTLs overlapping in OA-SA reached saturation at a much slower rate. In contrast we didn't detect any saturation for the cis eQTLs overlapping in all three tissues, instead an even faster rise in their numbers as the slope increases with an increase in sample size (Figure 1b, ABC). Since only the number of cis eQTLs detected in all three tissues continues to increase with an increase in sample size while partially overlapping and non-overlapping cis eQTLs reached steady numbers, the slope for ABC will eventually reach a maximum value bound by 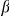 identified for a single tissue, as evident in Figure 1b.

For each set of cis eQTLs we then investigated if δ (the eSNP position relative to the start site of the transcript) was significantly changed with sample size (Figure 1c). On average, the cis eQTLs overlapping all three tissues had mean distance (the average is performed over all cis eQTLs in the set) *<δ>* significantly smaller than in any other set of cis eQTLs (see Figure S4 for box-plots of *δ*), while the tissue specific cis eQTLs displayed the largest average distance (the Mann–Whitney *U* test p values testing the difference between these 2 sets approximates 0 for any *i*). At the same time the average association strength measured as 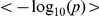 for each set is always highest for the overlapping cis eQTLs when compared to unique cis eQTLs (Figure 1d). This behavior has been observed previously in both mouse [17] and human studies [5]. One argument for this observed behavior [5] is that it's related to the intrinsic biology, with distal regulatory elements (regulatory enhancers) being more cell type specific than basic regulatory elements (characterized by those eQTLs detected in all tissues).

It has been reported that 54%, 50% and 54% of cis eQTLs in fibroblasts, LCLs and T cells respectively will be cell type specific [5]. However, our analysis using tissues collected from a human cohort shows that the number of unique cis eQTLs detected saturates quite rapidly as a function of sample size, while the total number of cis eQTLs common to all tissues continues to increase. Therefore, the percentage of common cis eQTLs is strongly dependent on the sample size. For example, the percentages of cis eQTL specific to OA, SA, and liver when the sample size is *n_1_* = 50, which is the minimum sample size we tested, are 42.8%, 38.4% and 55.9%, respectively. These percentages dramatically decrease to 23.5%, 21.1% and 35.6%, respectively, when the maximum sample size was tested, *N_20_* = 393. Because the cis eQTL common to all tissues show no sign of saturating, we expect these percentages to further decrease. In contrast the number of cis eQTLs overlapping all three tissues increased from 22.25%, 24.52% 32.19% in *N_1_* to 40.77%, 42.86%, 48.95% in *N_20_* in OA, SA and liver respectively. The opposite conclusion was reached in a previous report on fibroblasts, LCLs, and T cells likely due to three factors: 1) sample size, 2) homogenous cell population and 3) similarities/dissimilarities between the samples (tissues versus cells) compared.

**Figure 2:**
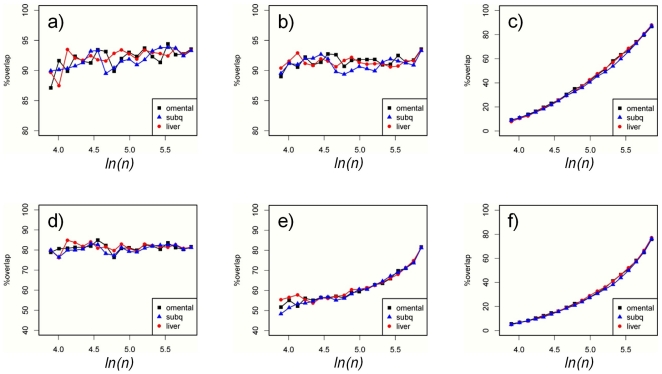
Single tissue analysis of cis eQTLs. **a**) Percentage overlaps between cis eQTLs detected in 2 consecutive sub samples. Approximately 90% of the cis eQTLs are found in common between consecutive sub samples. **b**) Percentage overlap between the cis eQTLs detected in a sub sample and the set of cis eQTLs detected in sample *N_20_*. As in panel **a**) we see more than 90% overlap between the cis eQTLs in any sub sample and the ones identified in the largest sample. **c**) Percentage of the cis eQTLs identified in *N_20_* represented in each sub sample. This plot is complementary to the plot in panel (**b**) the reference in this plot is the set of cis eQTLs detected in *N_20_*. While indeed 90% of cis eQTLs from smallest sub sample are recovered in the largest sub sample (**b**) they only represent about 10% of all cis eQTLs detected in *N_20_*. In panels **d–f** we plot the same quantities as in **a–c** but for the eSNPs. The set of eSNPs overlap at a rate of 80% between consecutive steps of the analysis as seen in panel **d**. **e**) In contrast to the high degree (∼90%) of overlap between cis eQTLs detected in any sub samples and the set of cis eQTLs in *N_20_* we see that replication for eSNPs is strongly affected by sample size, increasing from ∼50% to ∼80%. **f**) Relative to the eSNPs identified in *N_20_* we see a lower overlapping rate in sub samples than cis eQTLs.

Although we have already characterized the number of cis eQTLs detected as a function of sample size, a key question to address is whether the set of cis eQTLs and eSNPs identified in a specific tissue in smaller samples continue to be present in subsequent analysis as the cohort size is increased. To address this question we looked at the relative overlap between consecutive steps and plotted the percentage of cis eQTLs overlapping in 2 consecutive steps 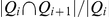. A very high rate of 90%, with approximately same value between any consecutive steps was observed (Figure 2a). Overlaps between cis eQTL sets relative to the largest sub sample *N_20_* defined as 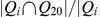 (Figure 2b) shows that more than 90% of the cis eQTLs identified in any sub-sample will be detected in the largest population. To gauge how many of the cis eQTLs identified in *N_20_* were also identified in sub sample *N_i_* we plotted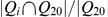 (Figure 2c). We found that approximately 10% of all cis eQTLs detected from *N_20_* were also detected in *N_1_* increasing to ∼90% in *N_19_* (similar plots as in Figure 2a–c for the sets of cis eQTLs previously described in Figure 1 can be found in Figure S3). Similar results for eSNPs are plotted in Figure 2d–f. We observed an approximate 80% overlap between eSNPs in consecutive steps 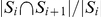 (Figure 2d), and an increase from ∼50% at (*i* = 1) to ∼80% at (*i* = 19) in overlapping eSNPs to the *N_20_*, 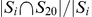 (Figure 2e). The percentage of eSNPs identified in the *N_20_* also identified in *N_i_*, 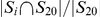 (Figure 2f), increases at a slower pace overall at the same sample size as shown for cis eQTLs. Overall, detection of cis eQTLs is much more robust than eSNP identification with about 90% overlap between consecutive steps for cis eQTLs and ∼80% for the eSNP. As additional evidence approximately 70% of cis eQTL and 50% of eSNPs identified at *N_20_* were also identified in the largest population available (n = 569 liver, n = 744 OA, n = 612 SA). We anticipate that these results could reflect strong correlation between different SNPs, each of which can equally well act as a surrogate measure for the true causal *cis*-acting effect within the linkage disequilibrium (LD) block. While this behaviour might be considered problematic, we see that in our study a maximum of eight different SNPs were associated to the same transcript in the 20 different sample subsets from each tissue. We used *ADRA2B* in OA (Figure S6a) and *ARNT* in liver as examples (Figure S6b) to demonstrate the change in lead eSNP between different consecutive analysis steps. These two genes were found to have the largest number of lead eSNPs in our analysis. We believe that this phenomenon should not have a damaging effect on eQTL identification overall, but should be considered when interpreting lead eSNP data for gene expression traits.

**Figure 3:**
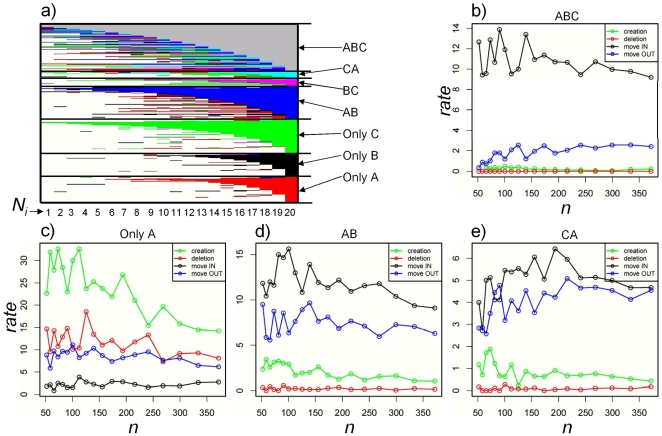
Cis eQTLs dynamics in the context of sample size for OA cis eQTLs. **a**) Cis eQTL overlaps (rows) in each sub sample (columns). If A,B,C represent OA, SA and liver, the color legend represents 7 classes of cis eQTLs as follows: red unique to OA, black unique to SA, green unique to liver, blue detected in both OA-SA, magenta detected in both SA-liver, turquoise detected in both liver-OA, grey common to all 3 tissues, white not detected in the analysis. We observe that new cis eQTLs at each step are first detected as nonoverlapping, specific to a tissue, while the majority of common cis eQTLs were previously detected in at least one tissue. Tuns as a function of sample size, a cis eQTLs will be first detected as unique to a tissue, than as sample size increases it will become detected in a pair of tissue or becoming common to all 3 tissues. **b–e**) Rate of change defined as change in number of cis eQTLs relative to change in sample size. We plot define four rates, relative to change in sample size: ***creation*** (green) defined as the number of new (not identified in a previous step in any tissue) cis eQTLs; ***deletion*** (red) defined as the number of cis eQTLs not detected in any tissue anymore from a previous step; ***move OUT*** (blue) defined as the number of cis eQTLs that do not satisfy the detection requirement of the class anymore; ***move IN*** (black) defined as the number of cis eQTLs that now satisfy the detection requirement of the class. **b**) cis eQTLs common to all 3 tissues have null creation and deletion rates indicating that a cis eQTL becomes common to all 3 tissues only if it was detected in a previous step. **c**) Unique to OA cis eQTLs have small move IN rates, with large creation rate. As sample size increases the creation rate approximates the sum of move OUT and deletion rates. Both partially overlapping cis eQTLs in OA-SA **d**) or liver-OA **e**) have approximate zero deletion and creation rates, with move IN rate larger that move OUT rate, the difference between these 2 rates becoming smaller as we increase the sample size.

**Figure 4:**
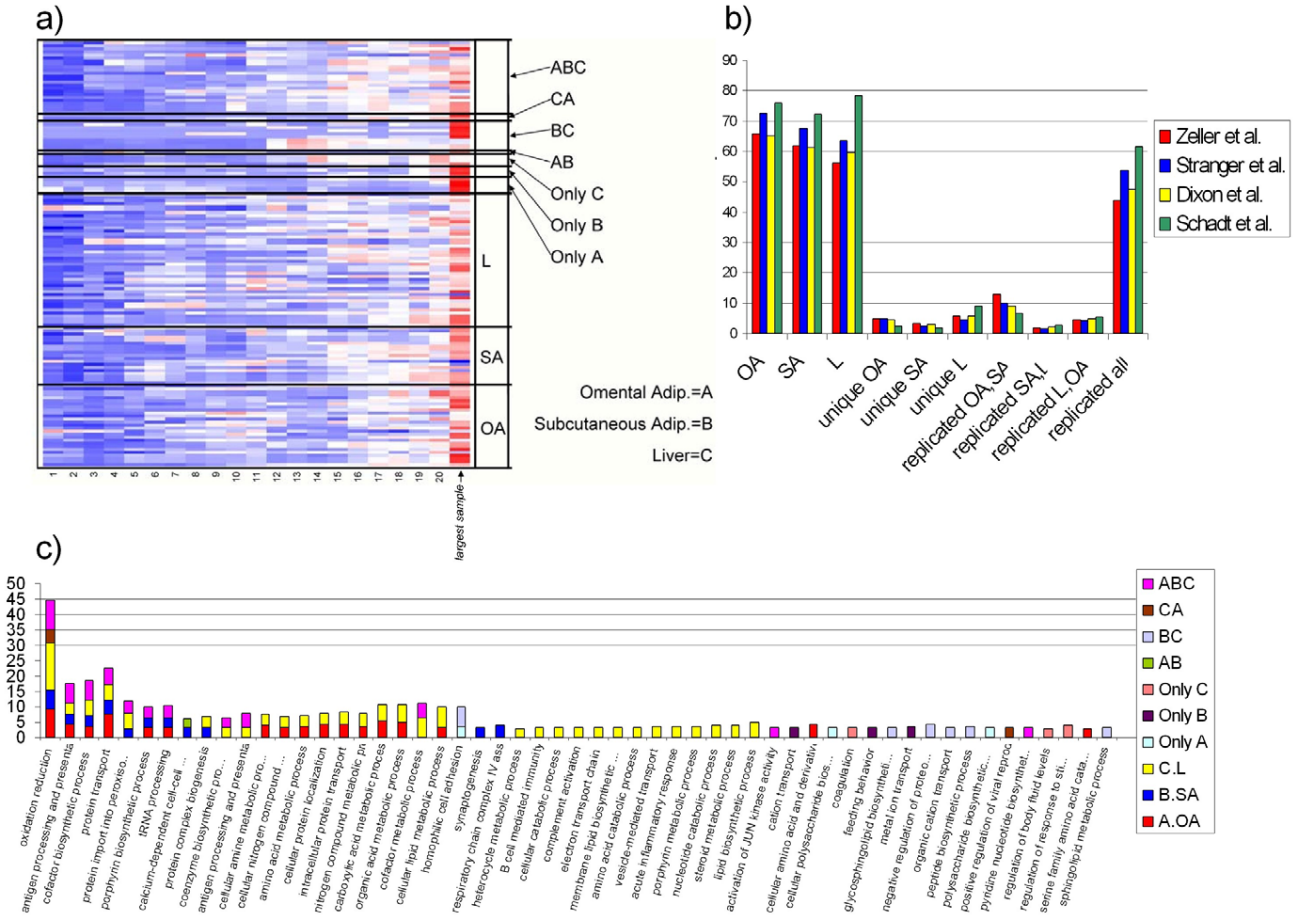
Gene Ontology (GO) enrichment for cis eQTLs. **a**) Heat map for GO Biological Processes terms that exceed FET p value 10^−3^ in the largest samples throughout the sub-sampling process. We see that p values decrease as sample size increases with a large jump from the 20^th^ sample to the largest sample. b) Cis eQTL replication in published studies. We observe an extreme large replication of published data in each of the tissues analyzed. Although published data does not replicate in the tissue specific cis eQTLs, we see a large overlap with the common cis eQTLs replicated in all the tissues in out analysis. **c**) Sacked bar-plot of 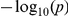 for a subset of GOBP terms using the largest samples. In terms of GO enrichment we see several terms that are enriched in each tissues individually as well as in the intersection. Liver cis eQTLs are also uniquely enriched for lipid processes as well as for immune response terms.

We have shown that the detection of cis-acting eQTLs obeys precise scaling laws in each tissue investigated. One important observation regarding replication of this effect is that the number of cis eQTLs present in all three tissues continues to grow with an increase in samples size while the number of tissue specific cis eQTLs starts to plateau with increasing sample size. This result is in contradiction to recent observations that approximately 50% of all cis eQTLs are tissue specific. The fact that the numbers of unique cis eQTLs are reaching a plateau could imply that we have already found a robust (i.e. that does not change with sample size) set of unique cis eQTLs. However if one looks at the strength of the association we find that unique cis eSNP are more weakly associated (higher p values) to gene expression traits than conserved cis eQTLs (Figure 1c) leading to a contradictory conclusion, as we would anticipate that the weaker unique cis eQTLs should contain a larger number of false positives compared to the set of universal and more strongly associated cis eQTLs. If we regard the increase in sample size as a dynamic change in the genetic information, a cis eQTL can be thought of as being created and then moving in the space between the 3 tissues, i.e. becoming detected in a pair of tissues or detected in all 3 tissues as well as being destroyed in the process of increasing sample size. In Figure 3a we plot a cis eQTL as it progresses from sub sample to sub sample. It is evident that an eQTLs can be viewed as a “dynamic” entity that undergoes transitions with regard to its tissue of origin. We hypothesize that an eQTL's first occurrence is likely as a unique cis eQTLs that can either disappear completely or more likely as we increase sample size becomes common to a pair of tissues then progresses to become detected in all tissues. In order to obtain a qualitative description of the cis eQTL dynamics we introduced four rates relative to change in sample size between consecutive steps: creation rate defined as the number of new cis eQTLs identified between steps (also not identified in any tissue in a previous step), deletion rate defined as the number of cis eQTLs lost between consecutive steps (no longer detected in any tissue), move OUT rate defined as the number of cis eQTLs detected in a particular combination of tissues that don't satisfy the requirement anymore and move IN rate, that is the opposite to move OUT rate and describes the dynamic of cis eQTLs that now satisfy a new requirement. In Figure 3b–e we plot these rates for cis eQTLs identified in OA tissue (similar results were observed for all 3 tissues), for the set of cis eQTLs detected in all 3 tissues (Figure 3b), unique to OA (Figure 3c), detected in both OA-SA (Figure 3d) and detected in both liver-OA (Figure 3e). The set of cis eQTLs common to all 3 tissues (Figure 3b) have a zero creation rate, indicating that a cis eQTL has to be detected first in a tissue then detected in a pair of tissues before becomes detected in all 3 tissues, zero deletion rate indicating that these cis eQTLs were detected in at least one tissue at a prior step, and a move IN rate ∼4× larger than move OUT rate, indicating a further accumulation of cis eQTLs common to all 3 tissues as we increase sample size. Unique cis eQTLs detected in OA tissue display the largest creation rate, and a very small move IN rate. The plateauing observed in Figure 1b for the number of unique cis eQTLs in OA correlates with the fact that overall creation rate approximates the sum of deletion and move OUT rates (the move IN rate is negligible) that leads to an overall unchanged number of unique cis eQTLs but with a different composition in the set. As with cis eQTLs common to all 3 tissues the ones common to a pair of tissues (Figure 4d–e) have approximate zero deletion rate, however move IN and move OUT rates are becoming close in value as we increase sample size that again signals a plateauing in their numbers as observed in Figure 1b. Looking at similar quantities as in Figure 2a, in consecutive steps we see an almost 100% overlap between cis eQTLs common to all 3 tissues, a 60% overlap for unique cis eQTLs in OA or SA and 80% overlap for cis eQTLs in liver (Figure S2).

eSNPs have not been identified for every known transcript or different transcript isoform, and beyond tissue specific expression it is not known why an eSNP present in one tissue (cell type) may not be detected in another tissue. We performed gene ontology analysis on the tissue specific eQTLs and the ubiquitous (found in many tissues) eQTLs to determine if we could identify any relevant biology specific to each of the eQTL subsets (Figure 4c). We note that the cohort used in the present study was predominantly morbidly obese subjects, which may affect both the anatomical and physiological state of the adipose tissue and the liver. Using Gene Ontology Biological Processes (GOBP) we find that the ubiquitous eQTL subset was enriched for oxidative reduction (FET p = 3×10^−10^) and antigen processing and presentation (FET p = 3.4×10^−10^). eQTLs found in both OA and SA where enriched for cell-cell adhesion (FET p = 2.3×10^−5^) and glycosphingolipid biosynthetic processes (FET p = 0.00043). This is of interest given the extreme obesity of the study populations. These pathways have been associated with adipose tissue remodeling, vascularization and metabolism (mainly insulin sensitivity) through changes in body weight [18], [19]. Liver specific eQTLs were enriched for regulation of response to stimulus (FET p = 0.00017) and blood coagulation (FET p = 0.00078), while SA specific eQTLs were enriched for metal ion transport (FET p = 0.00029) and feeding behavior (FET p = 0.00042). OA specific eQTLs were enriched for cell-cell adhesion (FET p = 6.3×10^−5^). The gene ontologies identified for eQTLs from a given tissue, without taking into account tissue specificity were more representative of tissue type, for example liver eQTLs were found to be enriched for lipid metabolic process (FET p = 1.5×10^−7^).

## Discussion

Using sub-samples from a cohort of obese individuals that underwent RYGB we were able to derive scaling laws for the number of cis eQTLs detected as a function of sample size in three tissues. We show that saturation for eSNP identification in the largest population analyzed was not achieved. We predict that given a sufficiently large population it is likely that for each transcript and transcript isoform variation we will be able to identify a corresponding regulatory variant (eSNP). Such a behavior is observed for the cis eQTLs overlapping all three tissues. In contrast, the cis eQTLs that overlap any two pairs of tissues or are unique to a tissue as they start to saturate for a given sample size. However the saturation seems to be on different scales in the two-tissue overlaps versus single tissue-specific cases. Overall we show that while there is no apparent slow down in the number of cis eQTLs detected in each tissue we observed that the detection of unique cis eQTLs for each tissue or found in pairs of tissues can be achieved much sooner.

As a dynamic quantity the cis eQTLs can become very stable if it becomes common to all 3 tissues, i.e. these cis eQTLs have zero deletion and creation rates. In contrast, unique cis eQTLs are the most unstable, dominated by a very high false discovery rate, ∼40% of unique cis eQTLs in OA or SA are lost in consecutive steps. Creation, destruction, move IN and move OUT rates explain the plateauing in the number of partially overlapping and unique cis eQTLs and the continuous rise in the number of common cis eQTLs. A key element in the analysis is tissue type. The two related tissues (OA and SA) share more cis eQTLs, with a weaker overlap with liver. The similarity between OA and SA tissues is responsible for the very large set of cis eQTLs found in common to both these tissues (that excludes cis eQTLs common to all 3 tissues).

Gene set analysis and replication efforts in existing studies help us understand and hypothesize the molecular mechanisms represented by different sets of eSNPs. cis eQTLs identified in all 3 tissues in our analysis prove to be the most stable, as the majority of them have been now replicated in four different studies [6], [20], [21], [22]. Unique cis eQTLs show a weak replication and also weak GO enrichment, possibly due to large false positive rates. The changes in enrichment with the increase in sample size did not provide additional information.

## Materials and Methods

### Data set description

Detailed information about the sample collection and data generation can be found here [1]. Briefly, tissues were collected from patients at the time of RYGB surgery at Massachusetts General Hospital between 2000 and 2007 as described previously [1]. Genomic DNA was extracted from liver tissues, total RNA was extracted from liver, SA and OA. RNA was converted to fluorescently labeled cRNA that was hybridized to custom 44K DNA oligonucleotide microarrays manufactured by Agilent Technologies as described previously [2], [23]. The custom array consists of 4,720 control probes and 39,820 non control oligonucleotides. Each DNA sample was genotyped on the Illumina 650Y BeadChip array. Sex was confirmed using PLINK [24]. Identity by state (IBS) analysis was performed to identify related individuals. Eighteen parent-offspring, 6 sibling and 8 second degree relatives were identified, 4 of these were related trios. 28 individuals were removed to eliminate IBS in the dataset. 393 subjects, which are self-reported Caucasians, were included in our analysis for which genotyping and gene expression data was available from all three tissues.

Demographic information including age, race, gender, height, type of surgery and year of surgery were collected for each patient. The cohort was predominantly (88%) self reported ‘white’ and female (75%).

### Microarray Data

The expression data is publicly available at GEO super series Accession ID GSE24335 and was previously described in [1]. All microarray data utilized in this study is MIAME compliant. Genotyping data is available at dbGAP (https://dbgap.ncbi.nlm.nih.gov/) and all data is available upon request from Massachusetts General Hospital at http://www.samscores.org.

### eSNP identification

Cis and trans acting expression quantitative trait loci (eQTLs) were identified using a method similar to that previously described [3]. Briefly, eQTLs for gene expression traits were determined by identifying the SNP most strongly associated with each expression trait profiled on the array over all genotyped SNPs. A cis eQTL occurs if the SNP and the transcript are within 1 Mb from each other regardless of the transcriptional start or stop of the transcript. All other eQTLs that are not cis- are trans-eQTLs. Association was tested to all expression traits measured on the array. Only associations that exceed 10% FDR are reported. FDR was estimated by permuting expression data.

## Supporting Information

Figure S1 **eSNP and eQTL numbers as function of sample size.** In panels **a**) and **b**) we show the eSNPs as function of sample size in the log-normal space **a**) and linear space **b**); similar plot for the number of cis eQTLs in panels **c**) and **d**). The plateauing in the number of cis eQTLs partially overlapping and nonoverlapping can be much easier observed. Clearly only the number of cis eQTLs common to all 3 tissues is increasing as function of sample size.
(TIF)

Figure S2 **Slope for the number of cis eQTLs.** Slope is calculated using a window of **a**) 10 consecutive points and **b**) 15 consecutive points. In order to assess the changes in the cis eQTL numbers vs. sample size in log-normal space (Figure 1b and Figure S1c, d) we compute slope in a window containing 10 or 15 consecutive points. We see that slope is increasing for cis eQTLs overlapping in all 3 tissues, with the rate of identifying non-overlapping cis eQTLs shows a decrease as we increase sample size.
(TIF)

Figure S3 **Detailed plots for cis eQTL analysis.** Similar to Figure 2a–c we plot in panel **a**) for each set of cis eQTLs between rate of conservation between consecutive steps, panel **b**) rate of overlap to cis eQTLs found in the largest sub sample and panel **c**) rate of overlap between cis eQTLs identified in the largest sub sample and cis eQTLs detected in the 20 consecutive sub-samples. Cis eQTLs common to all 3 tissues shows an almost 100% rate overlap between consecutive steps. Cis eQTLs unique to liver show a higher overlapping rate compared to cis eQTLs unique to OA or SA which we believe is due to tissue similarity. Similarly, cis eQTLs overlapping between OA and SA show a higher rate compared to any other pair of tissues.
(TIF)

Figure S4 **Box-plots for SNP-trait distance in** ***log_10_*** **space.** With red we marked overlapping cis eQTLs and white unique cis eQTLs in: **a**) OA, **b**) SA and **c**) liver. The reason we show distance *log_10_* transformed is due to the asymmetry of its distribution (heavily shifted to the left). Since the logarithm is a monotonic function the values for any non-parametric test remain unchanged. For each data point the p values for a parametric or non-parametric test between the groups yields values very close to 0 (the largest p value ∼10^−60^).
(TIF)

Figure S5 **Trend replication in different random sample series.** Total number of cis eQTLs in **a**) OA, **b**) SA and **c**) liver obtain in 5 different random realizations of the sub sample series. For 4 of the series we identified cis eQTLs in only the first 10 sub samples. The last data point is the total number of cis eQTLs detected with the largest sample available. We see that overall the inferred scaling laws are unchanged for any series.
(TIF)

Figure S6 **Sample specific eSNPs associated with** ***ADRA2B*** **and** ***ARNTL*** **.** We show an example 2 cis eQTLs, panel **a**) *ADRA2B* in omental adipose, and panel **b**) *ARNT* in liver, that are associated with different eSNPs while increasing sub sample size. On the positive side of the *y* axis we indicate the eSNP position and its strength of the association -*log_10_(p)* in each sub sample *i* (i.e. *i* takes values from 1 to 20, with *i* = 21 represents the eSNP generated in the largest sample), and the negative *y* axis are the *log_10_(p)* for the KW test between the expression levels of the transcript and all the SNPs in the window using the largest sample available for each tissue. The genomic position is represented on Mb scale on the *x* axis. We show the transcript position using the orange bar. We can see that as more genetic information is available from the increase in sample size the position of the cis lead eSNP changes. We also show the LD structure (R^2^) for that window below, data obtained from HapMap phase 2 and 3 release 27.
(TIF)

Table S1 **Sample sizes and SNP summary.** Sample sizes for all 20 sub samples together with the SNPs that did not pass a 5% MAF. The 2 additional columns show the sample size in log-normal space, and *delta* the difference between 2 consecutive samples sizes in log-normal space.
(TIF)
