# Supplementary figures and images for: Dissecting Cis Regulation of Gene Expression in Human Metabolic Tissues
Source: PLoS One. 2011 Aug 31;6(8):e23480. doi: 10.1371/journal.pone.0023480 (PMC3166146; doi:10.1371/journal.pone.0023480)

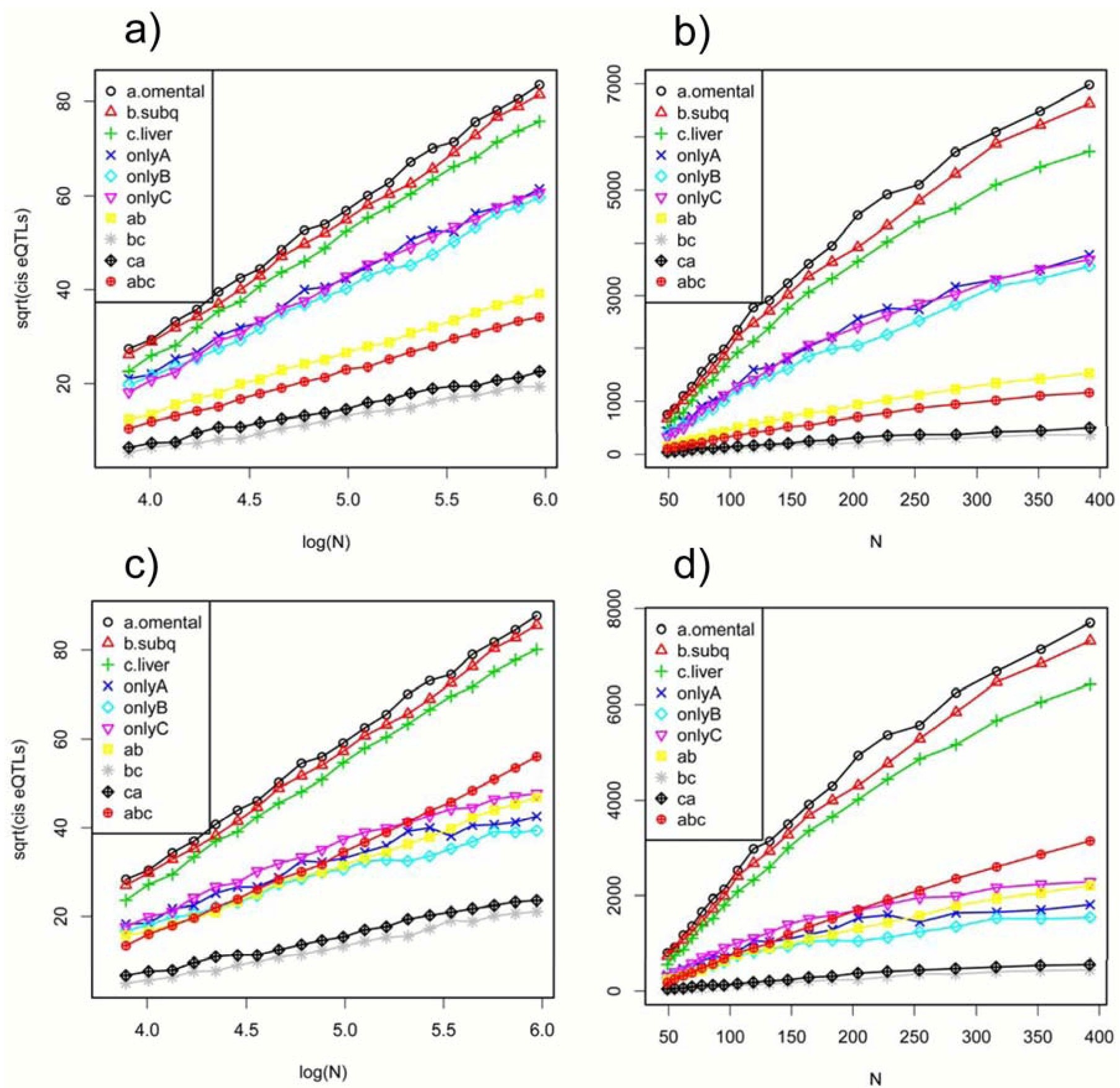

Supplement: Figure S1 — eSNP and eQTL numbers as function of sample size. In panels a) and b) we show the eSNPs as function of sample size in the log-normal space a) and linear space b); similar plot for the number of cis eQTLs in panels c) and d). The plateauing in the number of cis eQTLs partially overlapping and nonoverlapping can be much easier observed. Clearly only the number of cis eQTLs common to all 3 tissues is increasing as function of sample size. (TIF) [file pone.0023480.s001.tif]

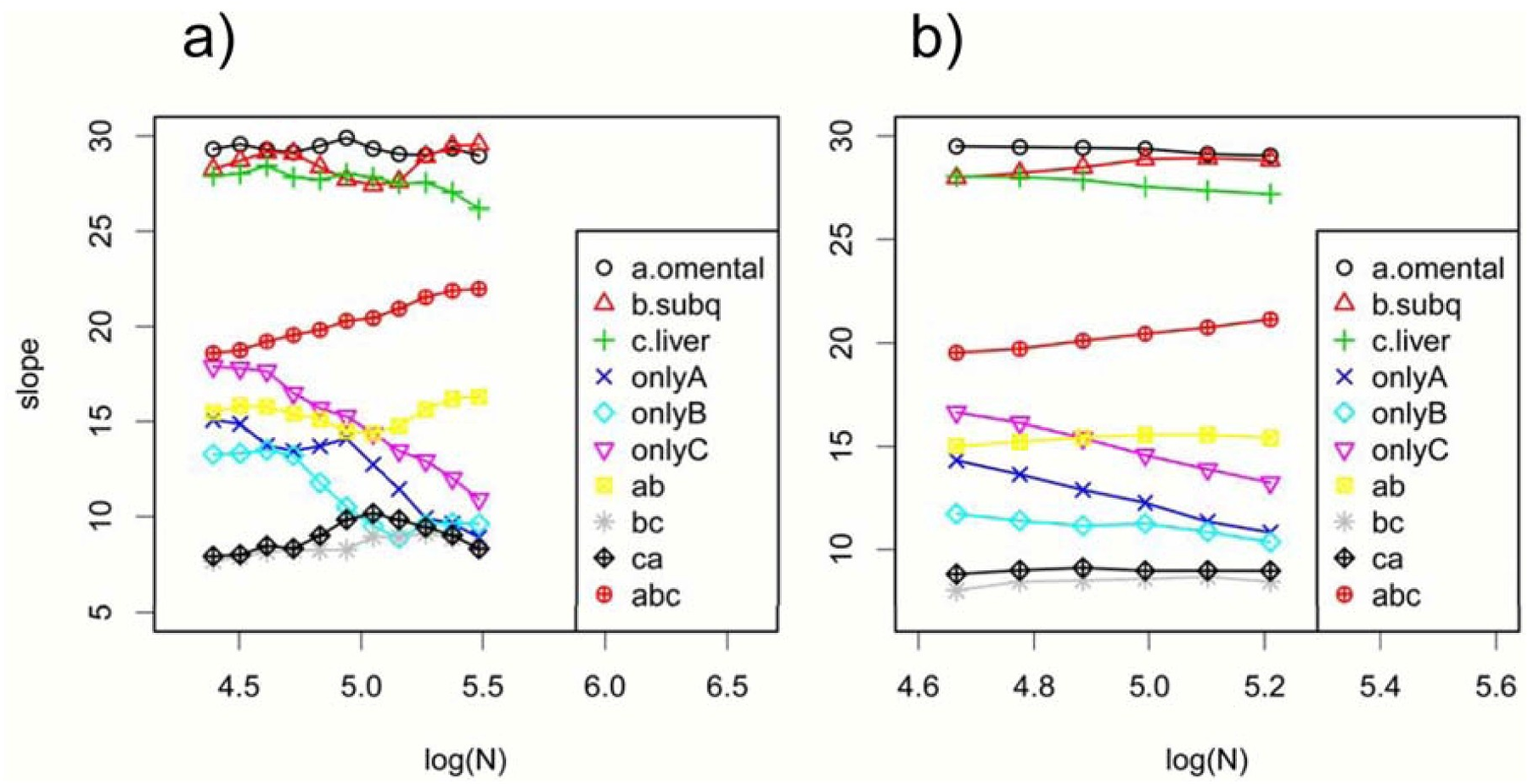

Supplement: Figure S2 — Slope for the number of cis eQTLs. Slope is calculated using a window of a) 10 consecutive points and b) 15 consecutive points. In order to assess the changes in the cis eQTL numbers vs. sample size in log-normal space (Figure 1b and Figure S1c, d) we compute slope in a window containing 10 or 15 consecutive points. We see that slope is increasing for cis eQTLs overlapping in all 3 tissues, with the rate of identifying non-overlapping cis eQTLs shows a decrease as we increase sample size. (TIF) [file pone.0023480.s002.tif]

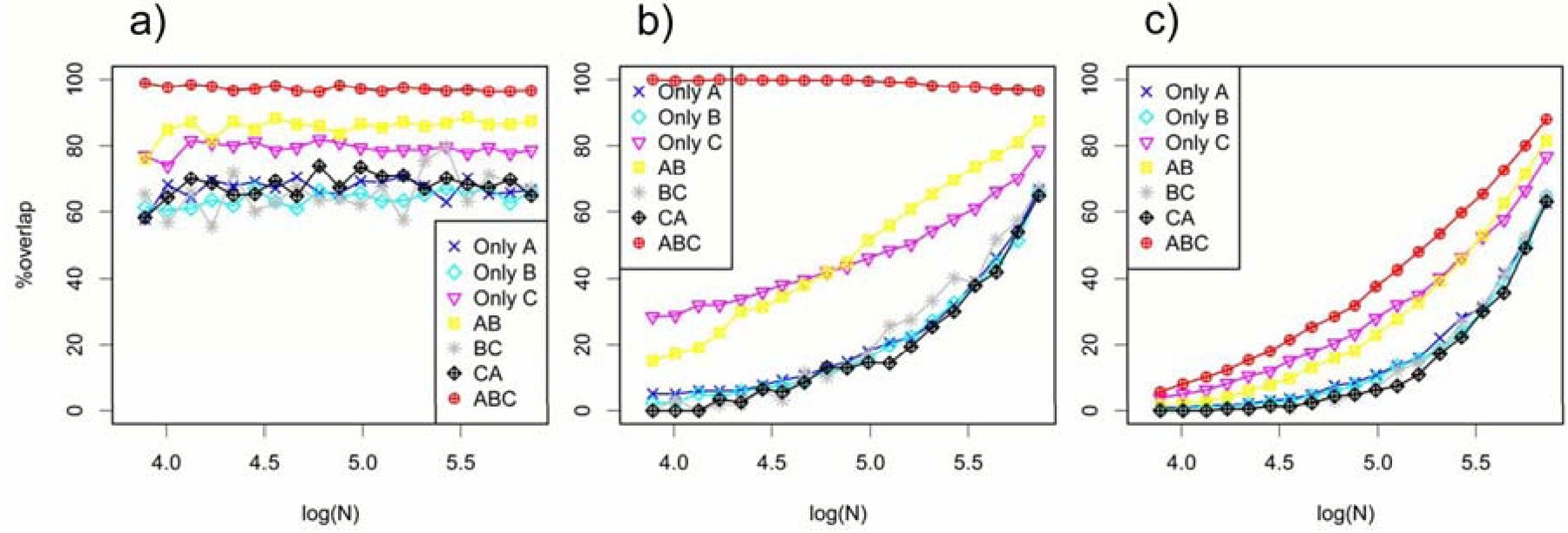

Supplement: Figure S3 — Detailed plots for cis eQTL analysis. Similar to Figure 2a–c we plot in panel a) for each set of cis eQTLs between rate of conservation between consecutive steps, panel b) rate of overlap to cis eQTLs found in the largest sub sample and panel c) rate of overlap between cis eQTLs identified in the largest sub sample and cis eQTLs detected in the 20 consecutive sub-samples. Cis eQTLs common to all 3 tissues shows an almost 100% rate overlap between consecutive steps. Cis eQTLs unique to liver show a higher overlapping rate compared to cis eQTLs unique to OA or SA which we believe is due to tissue similarity. Similarly, cis eQTLs overlapping between OA and SA show a higher rate compared to any other pair of tissues. (TIF) [file pone.0023480.s003.tif]

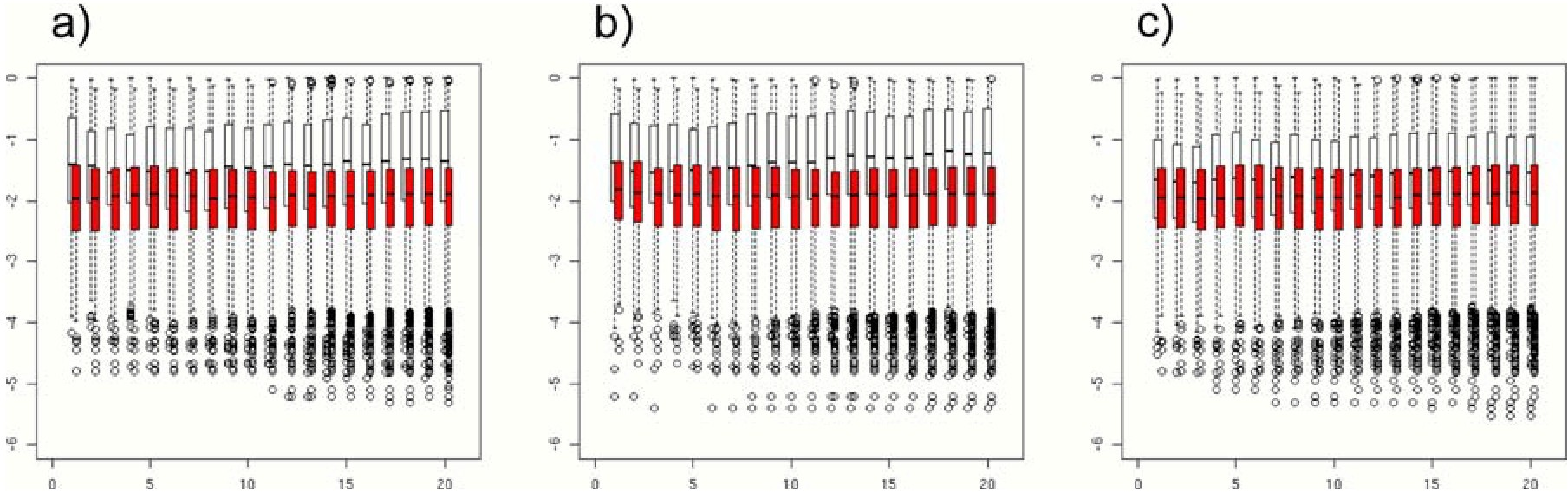

Supplement: Figure S4 — Box-plots for SNP-trait distance in log10 space. With red we marked overlapping cis eQTLs and white unique cis eQTLs in: a) OA, b) SA and c) liver. The reason we show distance log10 transformed is due to the asymmetry of its distribution (heavily shifted to the left). Since the logarithm is a monotonic function the values for any non-parametric test remain unchanged. For each data point the p values for a parametric or non-parametric test between the groups yields values very close to 0 (the largest p value ∼10−60). (TIF) [file pone.0023480.s004.tif]

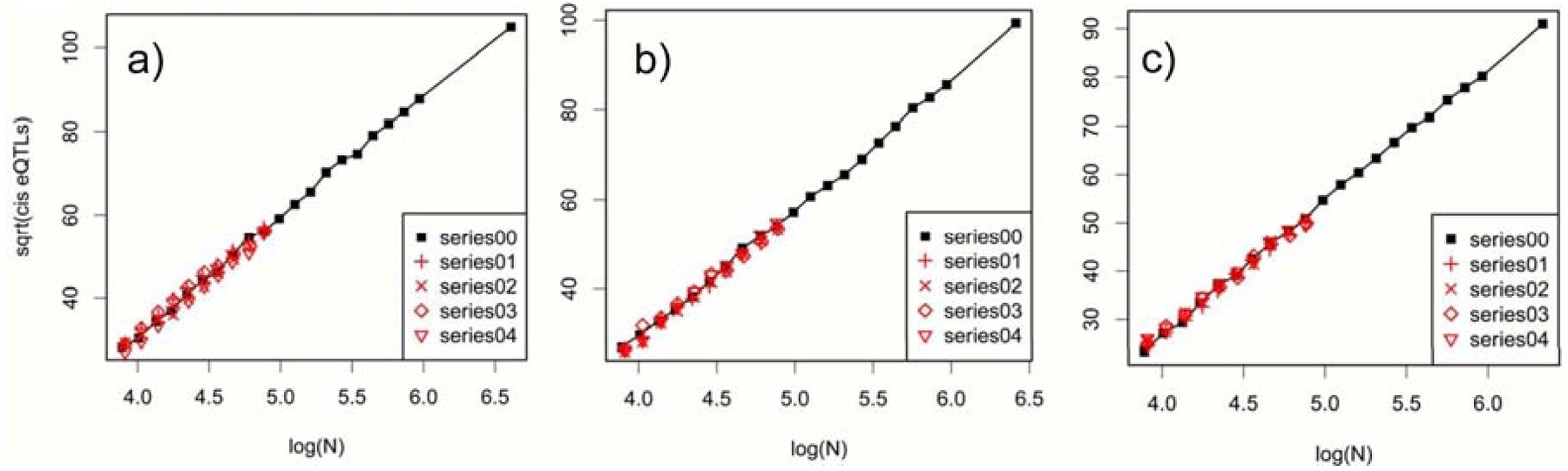

Supplement: Figure S5 — Trend replication in different random sample series. Total number of cis eQTLs in a) OA, b) SA and c) liver obtain in 5 different random realizations of the sub sample series. For 4 of the series we identified cis eQTLs in only the first 10 sub samples. The last data point is the total number of cis eQTLs detected with the largest sample available. We see that overall the inferred scaling laws are unchanged for any series. (TIF) [file pone.0023480.s005.tif]

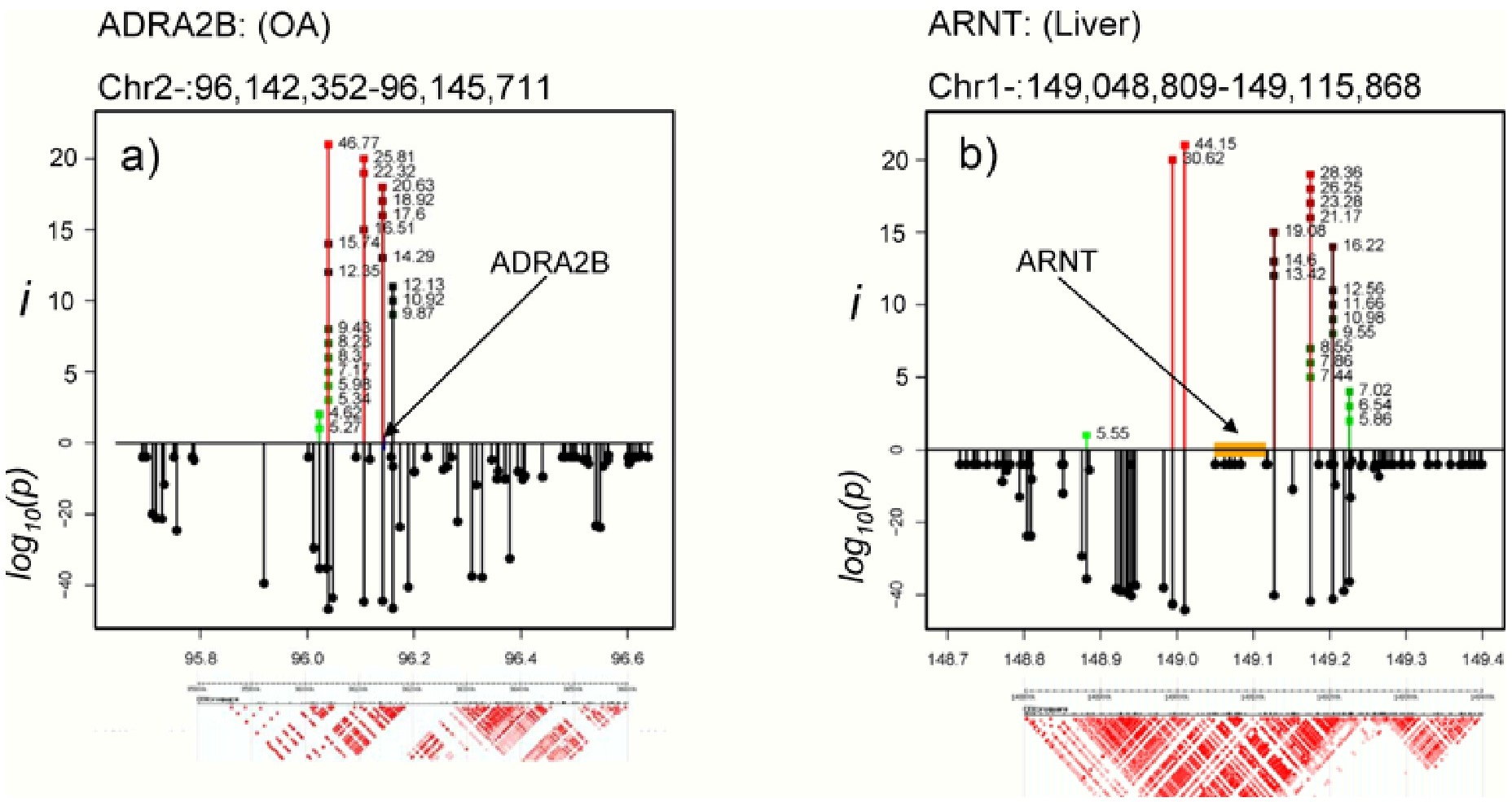

Supplement: Figure S6 — Sample specific eSNPs associated with ADRA2B and ARNTL . We show an example 2 cis eQTLs, panel a) ADRA2B in omental adipose, and panel b) ARNT in liver, that are associated with different eSNPs while increasing sub sample size. On the positive side of the y axis we indicate the eSNP position and its strength of the association -log10(p) in each sub sample i (i.e. i takes values from 1 to 20, with i = 21 represents the eSNP generated in the largest sample), and the negative y axis are the log10(p) for the KW test between the expression levels of the transcript and all the SNPs in the window using the largest sample available for each tissue. The genomic position is represented on Mb scale on the x axis. We show the transcript position using the orange bar. We can see that as more genetic information is available from the increase in sample size the position of the cis lead eSNP changes. We also show the LD structure (R2) for that window below, data obtained from HapMap phase 2 and 3 release 27. (TIF) [file pone.0023480.s006.tif]

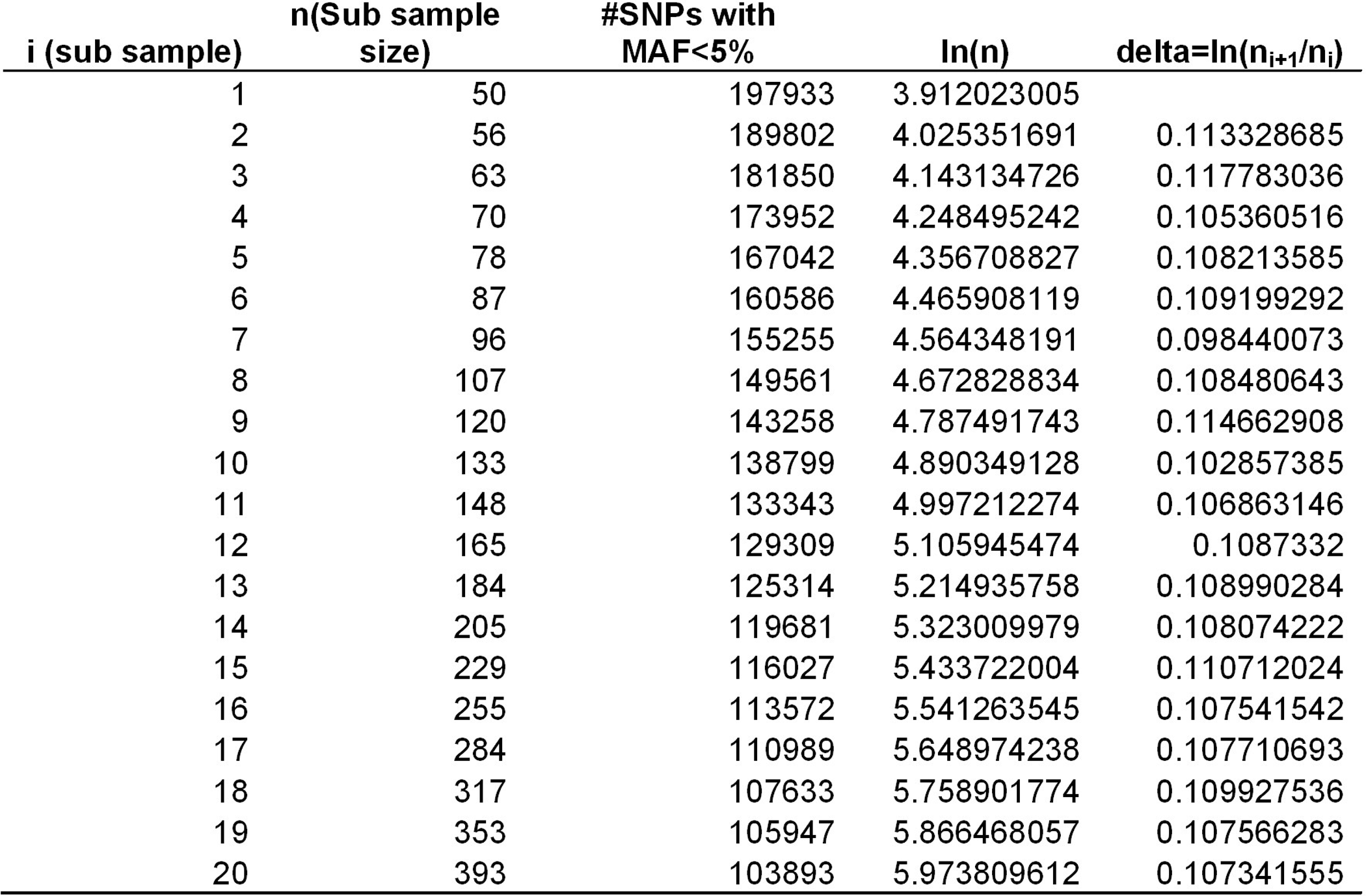

Supplement: Table S1 — Sample sizes and SNP summary. Sample sizes for all 20 sub samples together with the SNPs that did not pass a 5% MAF. The 2 additional columns show the sample size in log-normal space, and delta the difference between 2 consecutive samples sizes in log-normal space. (TIF) [file pone.0023480.s007.tif]
